# Discriminative validity of the Core outcome set functional independence in a population of older adults

**DOI:** 10.1186/s12877-020-01705-6

**Published:** 2020-08-26

**Authors:** Yvonne J. C. Dockx, Esther A. L. M. Molenaar, Di-Janne J. A. Barten, Cindy Veenhof

**Affiliations:** 1 Physical Therapy Sciences, program in clinical Health Sciences, University Medical Center Utrecht, Utrecht University, Utrecht, The Netherlands; 2 Department Innovation of Human Movement Care, Utrecht University of Applied Sciences, Utrecht, The Netherlands; 3 Department of Rehabilitation, Physiotherapy Science and Sport, UMC Utrecht Brain Center, Utrecht, The Netherlands

**Keywords:** Functional Independence, Core outcome set, Elderly, Validity

## Abstract

**Background:**

Clinicians are currently challenged to support older adults to maintain a certain level of Functional Independence (FI). FI is defined as “functioning physically safely and independent from another person, within one’s own context”. A Core Outcome Set was developed to measure FI. The purpose of this study was to assess discriminative validity of the Core Outcome Set FI (COSFI) in a population of Dutch older adults (≥ 65 years) with different levels of FI. Secondary objective was to assess to what extent the underlying domains ‘coping’, ‘empowerment’ and ‘health literacy’ contribute to the COSFI in addition to the domain ‘physical capacity’.

**Methods:**

A population of 200 community-dwelling older adults and older adults living in residential care facilities were evaluated by the COSFI. The COSFI contains measurements on the four domains of FI: physical capacity, coping, empowerment and health literacy. In line with the COSMIN Study Design checklist for Patient-reported outcome measurement instruments, predefined hypotheses regarding prediction accuracy and differences between three subgroups of FI were tested. Testing included ordinal logistic regression analysis, with main outcome prediction accuracy of the COSFI on a proxy indicator for FI.

**Results:**

Overall, the prediction accuracy of the COSFI was 68%. For older adults living at home and depending on help in (i)ADL, prediction accuracy was 58%. 60% of the preset hypotheses were confirmed. Only physical capacity measured with Short Physical Performance Battery was significantly associated with group membership. Adding health literacy with coping or empowerment to a model with physical capacity improved the model significantly (*p* < 0.01).

**Conclusions:**

The current composition of the COSFI, did not yet meet the COSMIN criteria for discriminative validity. However, with some adjustments, the COSFI potentially becomes a valuable instrument for clinical practice. Context-related factors, like the presence of a spouse, also may be a determining factor in this population. It is recommended to include context-related factors in further research on determining FI in subgroups of older people.

## Background

**Fig. 1:**
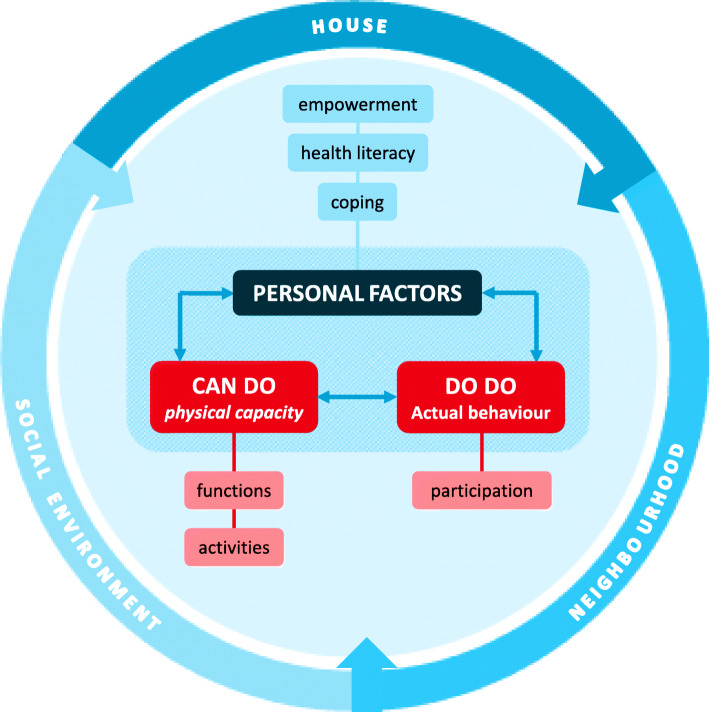
Graphical illustration of the concept of FI

When physical limitations increase, participating in activities of daily living becomes harder and living safely in the own home environment becomes challenging [1]. Problems in physical functioning potentially cause diminished self-sufficiency, loss of independence, and reduced quality of life [2]. Given the ageing society, which is commonly associated with an increase in physical limitations, one of the goals of the present healthcare system is to facilitate self-sufficiency and independent living as long as possible [3]. This is obviously reflected in clinical practice: clinicians are currently challenged to support older people in maintaining their ‘*Functional Independence’* [4, 5]. Previous studies on FI mainly focused on physical aspects when referring to FI (Molenaar et al. 2019, under review). However, ‘independence’ is a complex construct on itself, which certainly exceeds the physical domain. Baltes described the concept of ‘behavioral induced dependency’ in which environmental circumstances and especially social interactions are determinative for independent functioning [6]. There are several mechanisms explaining the development of dependent behaviors, assuming a learned dependency influenced by behaviors of caregivers. This implies independence in daily activities cannot be seen apart of independency of other people. A recent scoping review combining a literature review with expert consultations in the field of (community) care for older adults, confirmed the assumed wider look on the construct of FI, especially regarding the importance of older people’s context. In addition to the relevance of their social context, experts in the field of community care stressed the relevance of a safe home environment and neighborhood. Furthermore, literature suggests that coping style, health literacy and empowerment skills influence the level of FI in older people besides physical capacity [7–10]. Thus, physical, physiological and social aspects jointly represent the complex construct of FI. As a result, FI could be seen as a complex interaction between physical, psychological and social aspects respecting one’s home environment, social environment and neighborhood (Molenaar et al. 2019, under review). FI is defined as the ability of people to function physically safe and independent from another person, within their own context (Fig. 1) (Molenaar et al. 2019 under review).

A difference is expected in level of FI between people living in the community with and without support in daily activities and people living in a residential care facility. The main reason for admission to a residential care facility is the presence of substantial limitations in activities of daily living [3]. These are influenced by multi-morbidity, physical impairments, a low sense of self-management, and diminished social support [3]. A substantial part of this influencing factors is related to the concept of FI.

FI often exceed the borders of one specific profession; understanding, operationalizing and maintaining FI in older people calls for collaboration between various clinical expertises and underlying knowledge areas. To facilitate clinicians in adhering an unambiguous approach with respect to maintenance of FI in older adults, clinicians would increasingly benefit from an easy to use, interprofessionally interpretable instrument which supports them in precisely determining someone’s level of FI.

An instrument to objectify FI has recently been developed in a population of older adults: the Core Outcome Set Functional Independence (Molenaar et al. 2019, in progress). This Core Outcome Set theoretically comprises each of the domains of FI and is designed to assist clinicians in identifying, monitoring and supporting older adults who have (or are at risk for) limitations in FI. To enable clinicians to use the Core Outcome Set FI (COSFI) in clinical practice, the psychometric properties of the COSFI need to be assessed [11].

## Methods

### Aim

The primary objective of this study was to assess the discriminative validity of the COSFI in a population of Dutch, older adults (≥ 65 years of age) with different levels of FI. The secondary aim was to assess to what extent the non-physical related domains of the FI construct (coping, empowerment and health literacy) contributed to the classification in different levels of FI by the COSFI in addition to the domain ‘physical capacity’ in a population of Dutch older adults (≥ 65 years of age).

### Study design

This study was a cross-sectional validation study.

### Population and setting

Participants were community-dwelling older adults as well as older adults, living in residential care facilities in the Netherlands. Data were collected from February until May 2019. Identification of eligible older adults was done by district nurses and physical therapists. Also, recruitment took place by inviting older adults to participate through local newspapers and social media. Older adults could be included if they were 65 years or over and were able to understand verbal and written instructions in Dutch. Older adults with severe cognitive impairments which hindered completing the questionnaires, were excluded.

### Study procedure

Older adults were asked to come to a local test location or were offered a home visit to complete the measurements consisting of physical examinations and questionnaires. After giving written informed consent, they were guided through the approximately 60-min test-procedure. Physical examinations were conducted by trained researchers and students with different (clinical) expertise and background, for example physical therapy, occupational therapy and human movement sciences. Participants could complete the questionnaires themselves but were offered help from a member of the research team when needed. The test-procedure included (1) the measurements of the COSFI, (2) measurement of reference variable ‘membership of subgroup representing level of independence in daily living’ to validate the Core Outcome Set, and (3) a general questionnaire for demographic characteristics. The following section contains a description of the Core Outcome Set.

#### Measurements of the Core outcome set functional Independence

As no single instrument covered the total construct of FI, the COSFI was recently developed following recommendations from the Guideline for Selecting Outcome Measurement Instruments for Outcomes included in a Core Outcome Set [12]. The choice of specific measurement tools was determined by clinimetric properties of existing instruments representing the domains, their usability in the home-environment, availability in Dutch and multiple consensus meetings of the research group. Adjustments were made after pilot testing, based on the experiences of the researchers and the older adults who were tested.

The COSFI contains four domains:

- *Physical capacity*

Physical capacity was defined as the composite of all the physical capacities a person can draw on (generally described in terms of body system functions such as strength, balance) [13]. Physical capacity was measured by four physical tests. The *Short Physical Performance Battery* (SPPB) is recommended to assess physical capacity in older adults [14, 15]. The SPPB consists of three subscales: balance, gait speed and lower extremity strength [14, 15]. To test static balance more extensively, the *Frailty and Injuries Cooperative Studies of Intervention Techniques (FICSIT-study)* measurement instrument FICSIT-4 was added to the SPPB. It measures the ability to maintain balance over a diminishing base of support [16]. Because dynamic balance during walking is also an important component of physical capacity [17], the *Timed Up and Go test (TUG)* was added to the measurements. The TUG measures the time needed to stand up from a chair, walk three meters, turn, walk back and sit down. Furthermore, hand grip strength was measured three times for each hand with a *JAMAR hand-held dynamometer*, because this reflects overall muscle strength in older adults [18]. The maximum value in kilograms was administered [19].

- *Coping*

For coping the *Coping Flexibility questionnaire (COFLEX)* was used, based on the following definition of the domain: Ability of the individual to use both assimilative and accommodative coping strategies to deal with stressors in different situations (versatility and reflective coping) [20]. While using assimilative coping strategies a person actively influences his or her situation to reach personal goals. With accommodative coping strategies personal goals are tailored to restrictions of a given situation. The versatility scale measures the ability to switch between assimilative and accommodative coping strategies, depending on personal goals and environmental circumstances. The reflective coping scale measures the ability to choose a coping strategy that fits the circumstances. This results in two scores, one for each aspect. Both scales provide a number of statements with four answer possibilities: seldom or never, sometimes, often, almost always [20]. These correspond with scores of respectively one to four. The total scale-score is the sum of scores on that scale and a higher score indicates better coping skills. For versatility an example of a statement is ‘I can easily change my approach if necessary’ and for reflective coping ‘I question myself whether my approach to the problem is the best solution’.

- *Empowerment*

Empowerment was seen as the discovery and development of one’s inherent capacity to be responsible for one’s own life [21]. People are empowered when they have sufficient knowledge to make rational decisions, sufficient control and resources to implement their decisions, and sufficient experience to evaluate the effectiveness of their decisions [21]. The *Patient Activation Measure (PAM)* is recommended to measure the concept of empowerment [22]. The PAM-13 consists of thirteen statements, for example ‘I am confident that I can take actions that will help prevent or minimize some symptoms or problems associated with my health condition’. These are scored on a four-point Likert scale ranging from ‘totally disagree’ to ‘totally agree’ and ‘non applicable’. Based on this an activation score is computed between 0 (no activation) and 100 (high activation). On this continuum four levels of patient activation can be distinguished [23]. These levels are ‘disengaged and overwhelmed’, ‘becoming aware, but still struggling’, ‘taking action and gaining control’, ‘maintaining behaviors and pushing further’.

- *Health literacy*

Health literacy was defined as people’s knowledge, motivation and competences to access, understand, appraise and apply health information [24]. This enables them to make judgements and take decisions in everyday life concerning healthcare, disease prevention and health promotion to maintain or improve quality of life during the life course. The *Newest Vital Sign (NVS-D)* is a six-question tool to assess one’s level of health literacy by determining an individual’s ability to find and interpret information on an ice-cream nutrition label [25]. One of the questions asks people to calculate how many ice-cream they can eat when there is a restriction on intake of calories. For each right answer one point is administered, resulting in a score between zero and six. Cut-off point for adequate health literacy is a score of four or more.

Clinimetric properties of the included measurement instruments are described in Table 1.

**Table 1:** Clinimetric properties Core Outcome Set Functional Independence

Domain	Instrument	Target population	Clinimetric properties
Physical capacity	SPPB	Older adults [14]	Predictive for developing disability and identifies subgroups who have high and low risk of disability (AUC .75) [14, 26]. Cut-off points are scores of four and nine [27]. Good intrarater reliability (ICC .88–.92) and high construct and concurrent validity [14].
	FICSIT-4	Older adults [16]	Moderate to good reliability (Interclass Pearson correlations .25 to .74) [16]. Good concurrent validity [16].
	TUG	People with hip and knee osteoarthritis, patients with stroke and older adults with dementia [28, 29]	Good reliability (ICC .75 to .99) [28, 29]. Good construct and convergent validity [28, 30]. The cut-off point for independent walking is 20 s [31]. When it is impossible to complete the TUG, a fictitious score of 240 s is registered [31].
	JAMAR	General population and community-dwelling older adults [32, 33]	Excellent intra- and interrater reliability (ICC .98 and .94) [32]. Good test-retest reliability (ICC .91 for right and .95 for left hand) [33]. MCID is 6.5 kg [34].
Coping	COFLEX	Patients with chronic reumatoïd arthritis [20]	Acceptable internal consistency (Crohnbach’s α of respectively .88 and .70 for the subscales) [20]. Construct validity good for the versatility scale [20].
Empowerment	PAM-13	Older adults and older adults with multimorbidity	Good internal consistency (Crohnbach’s α of .88) [23, 35]. Good construct validity [35, 36].
Health literacy	NVS-D	Older adults	Good internal consistency (Chronbach’s α of .76) [25]. Cut-off point between adequate and inadequate health literacy is a score of four or more [25].

*SPPB* Short Physical Performance Battery; *FICSIT* Frailty and Injuries Cooperative Studies of Intervention Techniques; *TUG* Timed Up and Go test; *COFLEX* Coping Flexibility questionnaire; *PAM* Patient Activation Measure; *NVS-D* Dutch Newest Vital Sign; *AUC* Area Under the Curve; *ICC* Intraclass Correlation Coefficient; *MCID* Minimal Clinically Important Difference

### Outcome

Discriminative validity of the COSFI was determined following the COSMIN Study Design checklist for Patient-reported outcome measurement instruments [37]. Respecting this checklist, discriminative validity of the COSFI implies the degree to which the scores on the COSFI are consistent with hypotheses with regard to differences between relevant subgroups, based on the assumption that the COSFI validly measures the construct of FI [38]. With 75% of hypotheses reached, discriminative validity would be considered as confirmed [39].In this study, three distinctive subgroups were compared which differed in level of independence in daily living: community-dwelling older adults independent in (i)ADL, community-dwelling older adults, dependent on help of others in (i)ADL and older adults living in a residential care facility (Fig. 1). The subgroups were composed by way of a proxy indicator, based on two conditions. First, as the definition of FI includes ‘independent from another person’, help needed in (instrumental) activities of daily living ((i)ADL) was included in the proxy indicator. This was determined by the *Groningen Activity Restriction Scale (GARS-3)*, since GARS-3 showed adequate discriminative validity in a population of older adults [40]. This eighteen item questionnaire gives an indication of disabilities in the domains of personal care and domestic activities by registering if a person can do activities in three categories: fully independently with no effort (score one), fully independently with effort (score two) or only with help of others (score three) [40]. Any score on the GARS-3 other than ‘fully independently’ represented a diminished level of independency [40]. Second, as the definition of FI includes ‘functioning physically safe, within the own context’, *living situation* was part of the proxy indicator. When not functioning physically safe, falls are more likely to occur. Falls are a strong predictor for dependence in ADL-activities and for admission to a residential care facility [41–43]. A difference was made between living in the community (own context) and living in a residential care facility.

The following hypotheses were set regarding COSFI scores in relation to the distinctive subgroups of independence:

1. The model based on the COSFI correctly classifies at least 70% of the participants for each of the three subgroups.
2. There is a significant difference between groups on all domains of the COSFI with best scores for the group of older adults living in the community without help in (i)ADL and worst scores for the group of older adults living in a residential care facility.
3. Meaningful differences between these groups were expected on the scores of the measurement instruments in the physical domain of the COSFI, since it is known there is a difference in the amount of physical limitations between older adults living in their own environment and older adults living in a residential care facility [3]. For SPPB older adults living in their own environment were expected to score above the cut-off point of 9 and older adults living in a residential care facility below. For TUG older adults living in their own environment were expected to score below the cut-off point of 20 s and older adults living in a residential care facility above. For JAMAR a difference of at least 6.5 kg was expected between older adults living in their own environment and older adults living in a residential care facility.
4. A meaningful difference, reflected by a different level of patient activation on the PAM, was expected on the domain of empowerment between older adults living in their own environment and older adults living in a residential care facility. It is known that a low sense of self-management is related to admission to a residential care facility [3] and the concept of self-management is part of the definition of empowerment.
5. It was expected that the level of health literacy would be highest in the group of older adults, living in their own environment and independent of others (on or above the cut-off score of 4) and lowest in the group of older adults living in a residential care facility (below the cut-off score of 4), because literature shows people with lower health literacy skills show poorer overall health status [44].

### Statistical analyses

Descriptive statistical analyses were conducted on participant’s age, gender, educational level, presence of morbidities and type of residence. Demographic characteristics and scores on the Core Outcome Set were calculated by mean and standard deviation or median and interquartile range for continuous variables and proportions for categorical variables.

To test the formulated hypotheses determining discriminative validity of the COSFI several statistic techniques were used. The ability of the COSFI to predict the level of FI for three different subgroups was determined using ordinal logistic regression. First, the assumptions for logistic regression were tested. After that a model was built with group membership as dependent variable. All scores of the COSFI were entered as predictor variables in the ordinal regression model using ‘forced entry terms’. Based on the rule of thumb of ten events per variable for logistic regression analysis and eight included variables, a sample size of a minimum of 80 people in each group was optimal [45].

Differences between groups were calculated by One-way ANOVA for normally distributed continuous variables, Kruskal-Wallis for not normally distributed variables and Chi-square test for categorical variables.

To meet the second objective of the study, likelihood statistics of subsequently a null-model, a model with only physical capacity (SPPB, JAMAR) and models with physical capacity and respectively coping (COFLEX versatility, COFLEX reflective coping), empowerment (PAM) or health literacy (NVS-D) were tested for improvement of the model. Forward, stepwise ordinal regression analysis was applied, using a likelihood ratio (LR) test (X^2^ test) to determine to best fit. *P*-value < 0.05 was considered significant. When missing values occurred in the dependent or one of the predictor variables, these cases were excluded from the analysis.

Statistical analyses were performed using IBM Statistical Package for the Social Sciences (SPSS, version 24,0 Armork, New York, USA).

## Results

### Population

In total, 200 older adults were recruited to participate in this study. Eight persons were excluded because of severe cognitive impairments. One person living alone in the community, did not complete the GARS questionnaire and therefore, could not be included in one of the groups. Thirteen older adults had missing values on predictor variables and were excluded from the analyses. Finally, a total of 178 persons were included for analyses. Demographic characteristics are presented in Table 2. Sixty-six persons lived independently without help in activities of daily living. In the second group, independently living older adults who were dependent on help in at least some daily activities, sixty-nine persons were included. Forty-three older adults living in a residential care facility became the third group. Mean age was 80.2 years. Age significantly differed between the three groups. Independently living people without help had significantly fewer medical conditions, compared to the other groups. Gender and educational level did not differ between groups.

**Table 2:** Demographic characteristics of older adults measured for Functional Independence

Participant Characteristics	Total		Independent living No help with (i)ADL		Independent living Help with (i)ADL		Living in residential care facility	
	***n*** = 178		***n*** = 66		***n*** = 69		***n*** = 43	
Age in years at study participation (mean, sd)	80.19 (8.05)		76.50 (6.40)*		80.77 (7.30)*		84.93 (8.85)*	
Gender (n, %)								
*male*	72	40.4	32	48.5	26	37.7	14	32.6
*female*	106	59.6	34	51.5	43	62.3	29	67.4
Educational level (n, %)								
*none*	1	0.6	1	1.5	0	0.0	0	0.0
*primary*	121	68.0	42	63.3	47	68.2	32	74.4
*secondary*	38	21.3	15	22.7	13	18.8	10	23.3
*tertiary*	18	10.1	8	12.1	9	13.0	1	2.3
Type of residence in case of living in the community (n,%)								
*alone*	80	59.3	39	59.1	41	59.4		*na*
*with other(s)*	55	40.7	27	40.9	28	40.6		*na*
No. of medical conditions (n, %)								
*none*	12	6.7	9*^,†^	13.6	2*	2.9	1^†^	2.3
*one or two*	73	41.1	38*^,†^	57.6	23*	33.3	12^†^	27.9
*three or more*	93	52.2	19*^,†^	28.8	44*	63.8	30^†^	69.8

*(i)ADL* (instrumental) activities of daily living; *n* number of participants; *sd* standard deviation; *no*. number; % = percentage; *,† = significant difference between groups *p* < 0

### Discriminative validity Core outcome set functional Independence

While testing the assumptions for ordinal logistic regression, the FICSIT-4 was excluded from the analysis due to multicollinearity. The TUG was also excluded from analyses, as fictitious scores of 240 s were assigned to people who were unable to perform the test. This resulted in unjustified outliers.

All remaining measurement instruments of the COSFI were included as predictor variables. Only physical capacity measured with the SPPB was significantly associated with group membership. The odds of classification into the group of community-dwelling older adults depending on help instead of the group of independent community-dwelling older adults and of classification into the group of older adults living in a residential care facility instead of community-dwelling older adults depending on help increased by a factor 1.64 for each point decline on the SPPB (Table 3).

**Table 3:** Ordinal logistic regression prediction on group membership Functional Independence

	Wald	p-value	OR	95% CI
Physical capacity				
*SPPB*	49.909	0.000	1.64	1.43–1.88
*JAMAR*	3.377	0.066	1.03	1.00–1.06
Coping				
*COFLEX versatility*	0.989	0.320	1.04	0.96–1.12
*COFLEX reflective coping*	0.000	0.983	1.00	0.88–1.14
Empowerment				
*PAM*	0.246	0.620	0.99	0.97–1.02
Health literacy				
*NVS-D*	2.619	0.106	1.03	0.97–1.36

*Wald* Wald statistic; *p p*-value; *OR* Odds Ratio; *CI* Confidence Interval; *SPPB* Short Physical Performance Battery; *TUG* Timed Up and Go test; *COFLEX* Coping Flexibility questionnaire; *PAM* Patient Activity Measure; *NVS-D* Dutch Newest Vital Sign

Table 4 shows that this final model was able to correctly classify 68% of the participants into one of the predefined groups. In the group of independently living older adults without help and the group of older adults living in a residential care facility more than 70% was classified correctly. A percentage of 58% correctly classified is shown in the older adults living at home, needing help with (i)ADL. As we did not reach a 70% prediction accuracy for all three groups, hypothesis 1 was rejected.

**Table 4:** Prediction accuracy of the model based on the Core Outcome Set

		Predicted		
	*Independent living; no help with (i)ADL (n)*	*Independent living; Help with (i)ADL (n)*	*Living in residential care facility (n)*	% correct
**Observed**				
*Independent living; no help with (i)ADL (n)*	48	18	0	73%
*Independent living; help with (i)ADL (n)*	18	40	11	58%
*Living in residential care facility*	2	9	32	74%
Overall percentage	38%	38%	24%	68%

*(i)ADL* (instrumental) Activities of Daily Living

Results on instruments of the Core Outcome Set are presented in Table 5, both for the total sample as well as for the different subgroups. Significant differences between all three groups were found on all instruments for physical capacity and health literacy. For COFLEX Versatility and PAM-13 a significant difference was found between both groups living independently and the group living in a residential care facility. This confirmed hypothesis 2 about significant differences between subgroups on all domains (Table 5).

**Table 5:** Scores of older adults on the Core Outcome Set Functional Independence

Measurement instrument	Total	Independent living No help with (i)ADL	Independent living Help with (i)ADL	Living in residential care facility
	***n*** = 178	***n*** = 66	***n*** = 69	***n*** = 43
Physical Capacity				
*SPPB (median, IQR)*	9 (6)	10.5 (3)*	8 (5)*	3 (4)*
*FICSIT-4 (median, IQR)*	18 (13)	23 (6)*	18 (10)*	5 (11)*
*TUG (median, IQR)‡*	10.19 (6.76)	8.50 (3.08)*	10.71 (5.53)*	22.43 (14.19)*
*not able to perform TUG (n)*	10	0	3	7
*JAMAR (median, IQR)*	28 (16.25)	30 (12.5)*	27 (13.5)*	18 (11)*
Coping				
*COFLEX Versatility (median, IQR)*	26 (8)	28 (9)*	26 (8)†	24 (7)*^,^†
*COFLEX Reflective coping (median, IQR)*	11 (4)	11.5 (3)	12 (4)	10 (4)
Empowerment				
*PAM-13 (median, IQR)*	63.1 (21.8)	67.8 (21.8)*	65.5 (18.2)†	58.1 (16.6)*^,^†
Health Literacy				
*NVS-D (median, IQR)*	2 (3)	4 (4)*	2 (4)*	1 (2)*

*n* number of participants; *(i)ADL* (instrumental) activities of daily living; *SPPB* Short Physical Performance Battery; *TUG* Timed Up and Go test; *COFLEX* Coping Flexibility questionnaire; *PAM* Patient Activation Measure; *NVS-D* Dutch Newest Vital Sign; *IQR* interquatile range; *sd* standard deviation; *,† = significant difference between groups *p* < 0.05; ‡ Median, IQR for group who completed TUG measurement

Finally, meaningful differences on the different measurement instruments were evaluated regarding the preset hypotheses. For SPPB, TUG and JAMAR in the domain of physical capacity, hypothesis 3 was confirmed. On the PAM all three groups showed the same level of patient activation, meaning hypothesis 4 was rejected. On NVS-D older adults living at home and independent from others scored highest (4) and older adults living in a residential care facility scored lowest (1), which confirmed hypothesis 5.

Overall 60% of our hypotheses were confirmed.

### Contribution of domains coping, empowerment and health literacy to the model

To meet the secondary objective of this study, the additional value of non-physical related domains to the FI-construct were investigated by model building. The best fitting model compared to a model with only physical capacity, was a model with health-literacy (LR 17.240; df 1) and coping (LR 21.736; df 2) or empowerment (LR 20.962; df 1) (*p* ≤ 0.01). Adding both empowerment and coping instead of only empowerment or coping did not show a significant difference. (See Additional file 1).

## Discussion

The aim of this study was to determine discriminative validity of the COSFI in a population of older adults. With 60% of the hypotheses confirmed, discriminative validity of the COSFI did not meet the COSMIN criteria yet. However, the model based on the COSFI had an overall prediction accuracy of 68%, which is fairly good. Independently living older adults who did not need help in (i)ADL-activities and older adults living in a residential care facility are even better distinguished by the COSFI (73% respectively 74%). The model performed less in the group of older adults living at home and dependent on help, despite the fact that significant differences between groups were shown on most variables. In this group the prediction accuracy was just 58%. This means the model has insufficient ability to distinguish between people living at home with help of others and people living at home completely independently or people living in a residential care facility.

The difference in prediction accuracy between groups can be explained by several reasons. First, the group of older adults living at home, dependent on help, represents a broad range of FI. In this category the dependency on help varied from just periodical help from a pedicure to almost complete help in all (i)ADL activities. Second, the role of an informal caregiver might have influenced the relationship between the COSFI and our proxy. People living at home and dependent on help were often supported by an informal caregiver. This informal caregiver, for instance a spouse, enables older people to maintain living in their home setting instead of being institutionalized. Thus, some people in the group of independently living older adults with help seem to function at a comparable level as people in one of the other groups [46]. Involvement of an informal care giver might be a precondition for living at home instead of in an assisted living facility.

Our study revealed that FI is mostly determined by physical capacity, measured with the SPPB. This is in line with existing knowledge on the relationship between independent functioning and physical capacity [13, 27, 47]. However, this study adds that other domains also contribute to the construct of FI. Based on the current composite of measurements, a model with physical capacity, coping and one of the domains health literacy or empowerment performed equally good as a model with all domains. This implies that one of the domains health literacy or empowerment can be removed from the COSFI without losing discriminative value. Despite the fact that the NVS-D did not show to be a discriminative factor in distinguishing someone’s level of FI, differences in health literacy levels between groups were both significant and clinically relevant [25]. Health literacy is known to be associated with age, physical activity and participating in social activities [48, 49]. In line with these results from earlier studies, the oldest group in this study showed the lowest health literacy skills. On empowerment, no clinically relevant differences between groups were found [50]. All scores belonged to the same level of patient activation and measurement of empowerment did not contribute significantly to a model with all other domains. Therefore, a final model including physical capacity, health literacy and coping seems to perform optimally. The hypothesis about meaningful differences between subgroups on the domain empowerment was not confirmed. However, this hypothesis seems no longer relevant, as the domain empowerment does not significantly contribute to the best fitting model. For the remaining hypotheses 75% was confirmed, indicating good discriminative validity of the COSFI without PAM.

Some strengths of this study could be mentioned. It was the first study which measured a multidimensional concept of FI in older adults. Prior studies were limited to only the physical aspect of FI. Moreover, since the COSFI is intended for interprofessional use in clinical practice, another strength of this study was the cooperation of professionals and students from different backgrounds in the measurement procedure. Third, to determine discriminative validity, older adults with a broad range in levels of physical functioning were measured. This was an adequate representation of the Dutch population of older adults [3]. Finally, the limited amount of missing data could be indicated as a strength of this study.

There were some limitations as well. First, we insufficiently took into account the gradually decline of the level of FI. Currently, we only determined strict cut-off points for the proxy of FI. These cut-off points divided the older adults in three groups, representing a different level of FI. As previously mentioned, the middle group represented a broad range of levels of physical functioning, varying from almost no help to almost complete help in (i)ADL. With a good prediction accuracy for the other groups, the model shows ability to distinguish between older adults with different levels of FI, provided that differences are large enough. Another limitation concerns the influence of environmental factors on one’s FI. Literature shows an association between social support and home or social participation, which is closely linked to the definition of FI [51, 52]. In particular the presence of an informal caregiver or another person who provides social support is key for living at home respectively living necessarily in a residential care facility [3, 53]. The amount of social participation and the diversity in social relations influence decline in functional ability, measured on items like walking outside and walking stairs [52]. Although these items were to some extent objectified by the GARS-3, the involvement of (informal) caregivers was insufficiently determined. Dependency can be caused by several mechanisms, involving the social environment and interventions aiming to reduce dependency should acknowledge this [6]. The behaviors of caregivers are essential to enhance or decline independency in older adults. (Informal) caregivers can cause dependency by underestimating what an older adult can do and provide overcare or they can support independency by adjusting their behavior to the needs and possibilities of the older adult. In other cases, older adults choose for compensation and selection of activities by themselves. In that case, they are able to complete activities, but focus on goals and activities with high priority, asking for help in activities with low priority and as a result become dependent on others. In this study, only the activities which older adults were able to do were measured and not the activities they were actually performing in daily life. Third, although patient reported outcome measures (PROMs) were the only feasible option to measure coping, empowerment and help needed in (i)ADL, the use of PROMs is accompanied by some limitations. PROMs provide subjective data and the outcome is influenced by how the participant perceives his or her situation [54]. The perceived level of coping, empowerment or help needed may not reflect the actual behavior of the participant. Also, these questionnaires were not feasible for older adults with severe cognitive impairments. Therefore, older adults with limitations in their cognitive functioning were excluded from this study. Finally, with 178 participants optimal sample size was not obtained. The difference between actual and optimal sample size is small and probably did not have large impact on the results, because the smallest group is still distinctive.

Prior to implementation in clinical practice, it is recommended to revise the COSFI to some extent. Based on our results, the FICSIT-4 can be removed from the Core Outcome Set because its’ high correlation with the SPPB. Despite significant differences between groups, the TUG was not included in the analysis. Because a relatively large group participants was not able to perform the TUG, another instrument to measure dynamic balance possibly would have been more appropriate. As dynamic balance control is an important component of physical capacity and an indicator for risk of falls, this seems relevant to measure additionally to the SPPB. For instance, the Floor Transfer Test may be a good alternative for TUG as well as SPPB, because of high correlation with both instruments [55]. Besides that, it is recommended to remove the PAM from the COSFI. Without the PAM, a large part of the definition of empowerment is still covered in this model as the PAM did not measure all aspects of empowerment. Empowerment aspects were also represented by the NVS-D and COFLEX, because the constructs of health literacy and coping are associated with empowerment [21, 56]. Furthermore, it seems important to incorporate the influence of (social) environmental factors in the COSFI, such as the presence of an informal caregiver who facilitates an older person in performing activities of (instrumental) daily living. Although people with severe cognitive impairments are more likely to lose self-sufficiency, the questionnaires of the COSFI were not feasible for them [57]. In clinical practice however, this is a relevant group of older adults. For future studies and use in clinical practice, it is recommended to assess which adjustments to the Core Outcome Set or measurement procedure can be made to include this vulnerable group.

## Conclusion

In this study discriminative validity of the COSFI was assessed in a population of Dutch, older adults (≥ 65 years of age) with different levels of FI. The current composition of the COSFI, did not yet meet the COSMIN criteria for discriminative validity. However, with some adjustments, the COSFI has the potential to become a valuable instrument for clinical practice. This study indicates that the COSFI is moderately able to discriminate older people with different levels of Functional Independence. For 68% of older adults, the prediction of group membership by the COSFI is accurate. The COSFI has limited ability to distinguish between older adults living at home and dependent on help in (i)ADL and other groups. It is recommended to preserve the domains ‘physical capacity’, ‘coping’ and ‘health literacy’ as core elements of the COSFI. Complementary, in further research a measurement of environmental factors should be incorporated in the COSFI to improve clinical relevance in daily practice.

## Supplementary information

**Additional file 1.** Forward, stepwise ordinal regression. Contribution of each of the domains physical capacity, coping, empowerment and health literacy to the model based on the COSFI, determined by forward, stepwise ordinal regression.

## Data Availability

The datasets used and analyzed during the current study are available from the corresponding author on reasonable request.

## Abbreviations

FI: Functional Independence
COSFI: Core Outcome Set Functional Independence
(i)ADL: (instrumental) Activities of Daily Living
SPPB: Short Physical Performance Battery
FICSIT: Frailty and Injuries Cooperative Studies of Intervention Techniques
TUG: Timed Up and Go test
COFLEX: Coping Flexibility questionnaire
PAM: Patient Activation Measure
NVS-D: Dutch Newest Vital Sign
GARS: Groningen Activity Restriction Scale
LR: Likelihood Ratio
df: Degrees of freedom
PROM: Patient Reported Outcome Measure
